# A systematic review of providers’ attitudes toward telemental health via videoconferencing

**DOI:** 10.1111/cpsp.12311

**Published:** 2020-01-06

**Authors:** Samantha L. Connolly, Christopher J. Miller, Jan A. Lindsay, Mark S. Bauer

**Affiliations:** 1 Center for Healthcare Organization & Implementation Research, VA Boston Healthcare System, Boston, Massachusetts; 2 Department of Psychiatry, Harvard Medical School, Boston, Massachusetts; 3 HSR&D Center for Innovations in Quality, Effectiveness and Safety, Michael E. DeBakey VA Medical Center, Houston, Texas; 4 Baylor College of Medicine, Houston, Texas; 5 South Central Mental Illness Research, Education and Clinical Center, Houston, Texas

**Keywords:** attitudes, clinical video teleconferencing, implementation, providers, technology, telehealth, telemental health, telepsychiatry, videoconferencing

## Abstract

Telemental health conducted via videoconferencing (TMH-V) has the potential to improve access to care, and providers’ attitudes toward this innovation play a crucial role in its uptake. This systematic review examined providers’ attitudes toward TMH-V through the lens of the unified theory of acceptance and use of technology (UTAUT). Findings suggest that providers have positive overall attitudes toward TMH-V despite describing multiple drawbacks. Therefore, the relative advantages of TMH-V, such as its ability to increase access to care, may outweigh its disadvantages, including technological problems, increased hassle, and perceptions of impersonality. Providers’ attitudes may also be related to their degree of prior TMH-V experience, and acceptance may increase with use. Limitations and implications of findings for implementation efforts are discussed.

## 1 | INTRODUCTION

Telemental health, in which mental health care is provided from a distance, encompasses a broad range of practices, including the use of videoconferencing, e-mail, remote monitoring devices, and smartphone applications (Grady et al., 2011; Hailey, Roine, & Ohinmaa, 2008). Telemental health conducted via videoconferencing, referred to hereafter as TMH-V, provides real-time mental health care directly to patients and is rapidly expanding across private and publicly funded health-care systems worldwide (Australian Government Department of Health, 2018; Godleski, Darkins, & Peters, 2012; Lacktman & Rosen, 2017; O’Gorman, Hogenbirk, & Warry, 2015). Psychotherapy, medication management, and assessment services can be delivered via TMH-V to patients located at remote clinics or directly to patients’ homes, improving access to care by reducing travel time and costs and providing specialty services to underserved locations (Fletcher et al., 2018; Hubley, Lynch, Schneck, Thomas, & Shore, 2016). TMH-V has shown robust clinical effectiveness in multiple trials, and outcomes were demonstrated as noninferior to in-person care in several studies (Bashshur, Shannon, Bashshur, & Yellowlees, 2016; Hilty et al., 2013; Hubley et al., 2016). Patients have reported high satisfaction with TMH-V overall, describing it as effective and efficient (Kruse et al., 2017).

The effectiveness and patient-level acceptance of TMH-V have been well-documented, and there is a growing body of literature examining characteristics of mental health providers using this technology in practice (Glueckauf et al., 2018). However, there has yet to be a systematic review of providers’ attitudes toward TMH-V. This represents a critical gap in the literature, given that providers are often the gatekeepers of health-care innovations (Brooks, Turvey, & Augusterfer, 2013; Whitten & Mackert, 2005). If providers do not support the use of TMH-V, uptake may stagnate at the clinic level and patients may be unable to access TMH-V care. TMH-V has the potential to both benefit and inconvenience providers, and these factors may differentially impact providers’ attitudes. For example, while TMH-V may make care more efficient and accessible and can increase providers’ flexibility, it may also present new challenges with regard to navigating new technologies, coordinating care, scheduling appointments, and developing rapport with patients (Brooks et al., 2013).

It is therefore important to examine factors contributing to providers’ attitudes toward TMH-V. The unified theory of acceptance and use of technology (UTAUT; Venkatesh, Morris, Davis, & Davis, 2003) is a comprehensive framework that identifies four primary constructs underlying individuals’ acceptance of a technological innovation: performance expectancy, effort expectancy, social influence, and facilitating conditions. Performance expectancy is the extent to which an individual believes that an innovation will be useful and will have a relative advantage over other systems. According to the UTAUT, performance expectancy is the strongest predictor of intention to use a technology. Effort expectancy is defined as the perceived ease of using a given innovation. Social influence refers to perceptions that important people, such as organizational leadership, want the individual to use a new technology, and would view the individual positively for doing so. Facilitating conditions are defined as the perceived level of organizational and technical infrastructure in place to support use of the technology. The UTAUT posits that these four constructs can be moderated to varying extents by user age, gender, and experience using the technology, as well as the degree of voluntariness of adopting the innovation.

The current systematic review had three primary aims. The first was to assess the extent to which providers’ overall attitudes toward TMH-V were positive or negative. The second aim was to situate these findings within the key constructs outlined in the UTAUT, namely the extent to which TMH-V was found to be useful, easy to use, encouraged by organizational leaders, and supported by necessary infrastructure. The third aim was to examine potential influences of provider age, gender, experience with TMH-V, and voluntariness of use on attitudes toward this innovation.

## 2 | METHODS

We conducted a comprehensive review of the Englishlanguage, peer-reviewed literature on providers’ attitudes to ward TMH-V, published between January 2000 and June 2019 within three databases: PubMed, CINAHL, and Embase. Each subsequent database search filtered out results from the previous databases to prevent duplicates. Our initial search strategy required articles to contain a term pertaining to TMH-V as well as a term referring to providers or attitudes/experiences within their titles and/or abstracts. The complete list of searched terms is as follows (with asterisks indicating that any words beginning with that string of letters were included):

- TMH-V terms: telemental health, tele-mental health, telepsychiatry, telepsychotherapy, telepsychology, teletherapy, e-therapy, online therapy, online counseling, OR [(telehealth, telemedicine, telecare, video*, communication tech*) AND (psych*, mental*)]
- Provider terms: provider*, clinician*, psychiatrist*, psychologist*, stakeholder*, nurse*, professional*, physician*, social work*, personnel*
- Attitude terms: attitude*, perception*, experience*, interest*, opinion*, view*, utilization, satisfaction

The first author screened the titles and abstracts of the articles resulting from this search and identified those appropriate for full-text review. To meet criteria for inclusion with this review, articles were required to include the following:

- Information regarding providers’ attitudes toward or experiences using TMH-V to provide direct, real-time clinical care to patients. Consulting with another provider by videoconference, or the use of alternative technologies such as secure messaging, store and forward communications, or web-based treatment protocols did not qualify.
- Information from the perspective of the provider who conducted/would be conducting the TMH-V session with the patient versus from a primary care or emergency room doctor requesting a TMH-V consult.
- Qualitative and/or quantitative data collected using an explicitly identified measure, such as a survey or semi-structured interview. Articles that did not indicate and describe the measure used to collect their data were excluded.
- At least four providers meeting the above criteria were sampled. Providers could be of varying disciplines. This number was chosen in an attempt to achieve balance between including as many high-quality studies as possible, while also helping ensure that the interpretations of findings were reasonably generalizable to the broader population of mental health providers.

### 2.1 | Reliability

The first author identified a subset of 10 articles from within the 86 identified for full-text review, some of which the first author had determined to meet inclusion criteria, others of which were ultimately excluded. These articles were independently scored as eligible or ineligible by co-authors CJM and MSB. Fleiss’s kappa for the three raters within this subset of manuscripts was .829, indicating excellent interrater reliability. SLC, CJM, and MSB then met for a consensus meeting in which the inclusion/exclusion of all 86 articles was discussed, guided by a document compiled by SLC including detailed summaries of the articles’ study designs, participants, and findings. The group reached consensus on the 38 articles included in the final sample.

### 2.2 | Analytic approach

The following information was reported for each included study: the number, discipline, and extent of TMH-V experience of providers sampled, the type of services provided through TMH-V, characteristics of the patient population, the location at which patients received TMH-V care, the measures used to assess providers’ attitudes, and a brief summary of main findings. TMH-V experience was defined as whether or not providers had ever conducted a TMH-V session with a patient. Risk for selection bias, performance bias, detection bias, attrition bias, and selective reporting bias was assessed for all studies according to Cochrane Collaboration guidelines (Higgins & Green, 2011).

At the broadest level, it was determined whether providers’ overall attitudes toward TMH-V were reported as primarily positive or negative within each study, when that information was available. Each article was then coded for the presence of the four primary UTAUT constructs within provider attitude data: Performance expectancy, effort expectancy, social influence, facilitating conditions, and subconstructs were identified. The frequency with which subconstructs were endorsed, and the percentage of all articles that included each subconstruct were then calculated, and findings were summarized across articles. Next, based on the UTAUT model, the potential moderating role of gender, age, experience, and voluntariness of use was considered within provider attitude data. Articles were coded as to whether they presented findings involving any of these moderators. Experience was the only moderator discussed across multiple articles; these results were summarized across articles. Additional distinctions in providers’ attitudes not encompassed in the above sections were then coded and summarized.

## 3 | RESULTS

**FIGURE 1:**
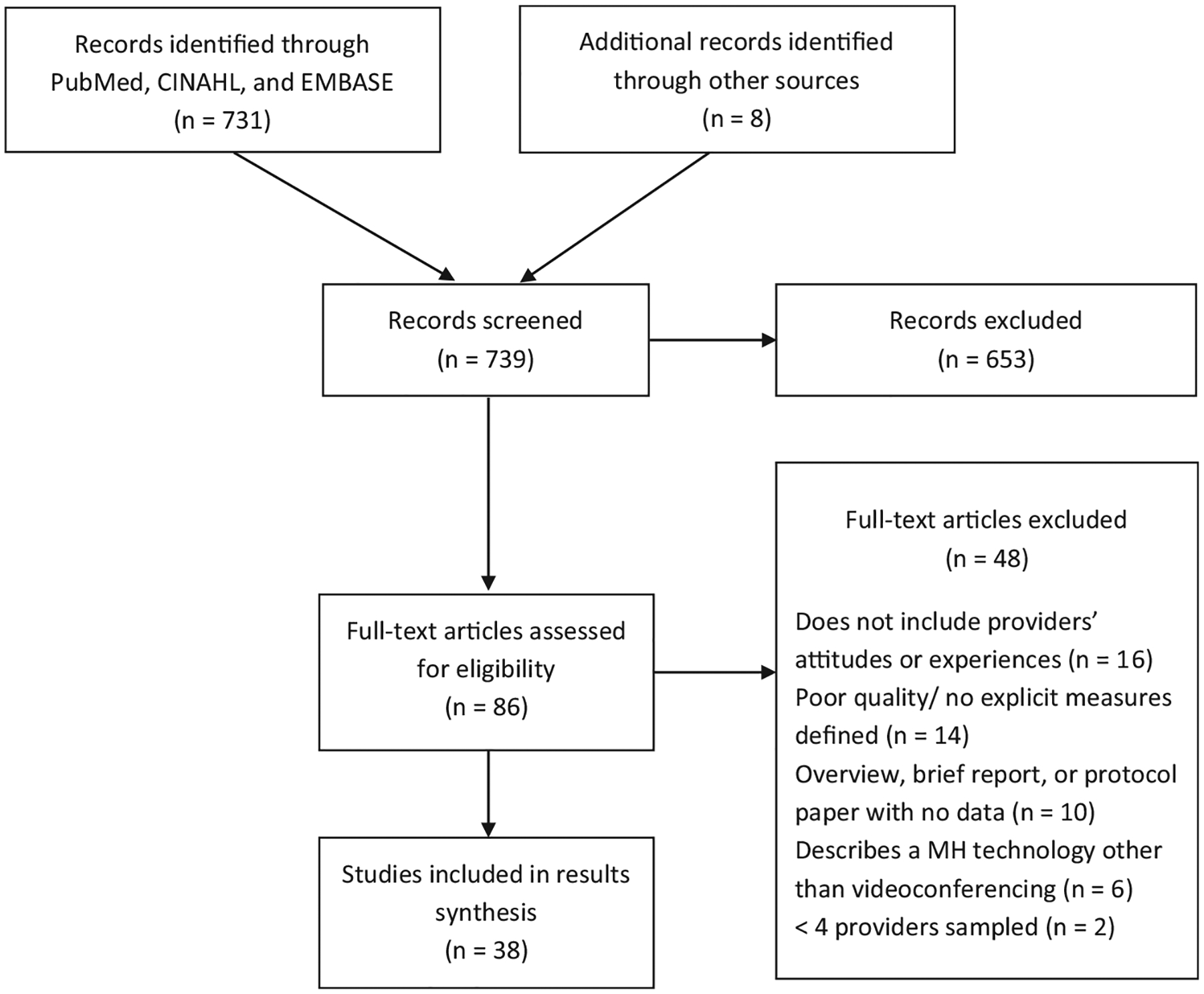
Modified PRISMA diagram

The initial search strategy resulted in 739 articles. Eightysix articles were identified as appropriate for full-text review after title and abstract screening (see modified PRISMA diagram, Figure 1). Of these 86 articles, 38 ultimately met criteria for inclusion with this review (Table 1). Twenty-eight studies (74%) assessed providers’ attitudes toward specific modes of TMH-V treatment, for which the patient population and the location of patients’ care were identified; the remaining ten studies (26%) were surveys of providers’ attitudes toward TMH-V more generally. Seventeen studies exclusively sampled providers with TMH-V experience (45%), 19 studies included providers both with and without experience (50%), and two studies exclusively sampled providers with no experience (5%). TMH-V was described as having various uses, including providing psychotherapy (22/38, 58%), short-term consultation and assessment (17/38, 45%), and medication management (12/35, 34%). Twenty-eight studies specified the locations at which patients received TMH-V care, including hospitals and clinics (21/28, 75%), within their homes (5/28, 18%), or in other locations, including schools, youth centers, crisis homes, or community centers (4/28, 14%). Fifteen studies involved the treatment of child and adolescent patients (39%), ten included military personnel/veterans (26%), and three included indigenous populations (8%). Eighteen studies (47%) were conducted outside of the United States, including Canada (7/38, 18%), Australia (5/38, 13%), and the United Kingdom (3/38, 8%). One study was conducted in each of the following countries: China, Finland, Italy, and Norway.

### 3.1 | Overall attitudes toward TMH-V

#### 3.1.1 | Positive attitudes

Overall attitudes toward TMH-V were largely positive (Adler, Pritchett, Kauth, & Nadorff, 2014; Baird, Whitney, & Caedo, 2018; Brooks, Manson, Bair, Dailey, & Shore, 2012; Cunningham, Connors, Lever, & Stephan, 2013; Levy & Strachan, 2013; Mitchell, MacLaren, Morton, & Carachi, 2009; Moreau et al., 2018; Whitten & Kuwahara, 2004; Wynn, Bergvik, Pettersen, & Fossum, 2012), such that providers were satisfied with TMH-V (Interian, King, St. Hill, Robinson, & Damschroder, 2017; Lindsay et al., 2017; Mayworm et al., 2019; Ruskin et al., 2004) and favorable toward its use (Cipolletta & Mocellin, 2018; Moreau et al., 2018), describing it as important (Glover, Williams, Hazlett, & Campbell, 2013; Volpe, Boydell, & Pignatiello, 2013) and an acceptable mode of treatment delivery (Elford et al., 2001, 2000; Thomas et al., 2017). These positive opinions were observed across types of services provided, location of TMH-V care, and patient populations.

Providers’ positive attitudes most frequently included reference to the UTAUT construct of performance expectancy, with multiple articles describing the innovation as being effective (Jameson, Farmer, Head, Fortney, & Teal, 2011; Perle, Burt, & Higgins, 2014; Starling & Foley, 2006) and useful (Gelber, 2001; Gibson, O’Donnell, Coulson, & Kakepetum-Scghultz, 2011; Glueckauf et al., 2018; Kopel, Nunn, & Dossetor, 2001; Simms, Gibson, & O’Donnell, 2011). One statistical analysis found that providers’ perception of usefulness was the most important factor in determining intention to use TMH-V based on questionnaire data (Monthuy-Blanc, Bouchard, Maïano, & Seguin, 2013), consistent with UTAUT hypotheses (Venkatesh et al., 2003). Six subconstructs of performance expectancy were identified (Table 2). The most commonly endorsed subconstruct was increasing access to care, such as for patients in remote locations, with disabilities or illnesses that made travel difficult, or with child or elder care responsibilities, as well as allowing for care during inclement weather (16/38, 42%; see Table 2 for included studies). The second most frequently endorsed benefit was saving time and money and increasing efficiency of services (12/38, 32%). Multiple articles also shared provider sentiments that TMH-V could have advantages over in-person care in some circumstances, such as by increasing patient comfort and decreasing inhibition when discussing sensitive subjects including trauma (8/38, 21%). Several of these advantages over in-person care were specific to providing TMH-V directly to patients’ homes versus to another clinic (Gordon, Wang, & Tune, 2015; Lindsay et al., 2017). These included allowing care to be received discreetly and privately at home, offering a window into a patient’s living environment, and serving as a stepping stone into care for avoidant patients. Receiving feedback that patients liked TMH-V was also noted as a positive aspect (6/38, 16%). TMH-V was described as providing increased flexibility, such as by allowing providers to work from different locations or check in more easily with high-risk patients (4/38, 11%). TMH-V was also mentioned as providing new job opportunities for providers, expanding the reach of their work, and facilitating increased collaboration with other clinicians (3/38, 8%).

Regarding effort expectancy, multiple articles reported that providers found TMH-V to be easy to use (7/38, 18%). The remaining two constructs were discussed less frequently. In terms of social influence, several articles described the positive impact of supportive leadership on providers’ attitudes toward TMH-V (3/38, 8%). Facilitating conditions, such as having strong technical support services, were also mentioned in several articles (4/38, 11%).

#### 3.1.2 | Negative attitudes

Although overall attitudes toward TMH-V were largely positive, there were several exceptions. A minority of psychiatrists (4 of 11) reported being satisfied providing medication management through TMH-V in one study (Wagnild, Leenknecht, & Zauher, 2006). They described technological and scheduling barriers, perceptions of TMH-V as impersonal, and reinforcement of patients’ social isolation as drawbacks. Two studies reported difficulty recruiting clinicians to provide TMH-V care (Adler et al., 2014; Shulman, John, & Kane, 2017). Reasons for providers opting to not participate included lack of interest (Adler et al., 2014) and concerns about technological issues (Shulman et al., 2017); both studies noted that perceptions of extra effort and hassle associated with TMH-V were deterrents.

Multiple drawbacks to TMH-V use were noted regarding performance expectancy, including articles that reported positive attitudes toward TMH-V overall. Six subconstructs were identified, and the frequency with which they were endorsed is reported in Table 3. Concerns were often raised regarding TMH-V being impersonal or interfering with the therapeutic relationship. Difficulties detecting nonverbal cues such as fidgeting or crying, poor hygiene, or signs of intoxication were noted, along with trouble maintaining eye contact and disruptions to conversation flow (19/38, 50%; see Table 3 for included studies). Safety and legal concerns were common, including the inability to be physically present to assess for risk and coordinate patient transfer to a hospital; some providers questioned their liability in the case of a crisis and the limitations of their licenses, such as whether they could legally see a patient via TMH-V across state lines (13/38, 34%). Some providers felt that patients would not like using the TMH-V modality as compared to in-person visits (8/38, 21%). A lack of provider knowledge regarding TMH-V security and confidentiality was noted in several articles (7/38, 18%). Providers also raised the possibility that certain patients, such as those with hearing or visual impairments, or those who are socially isolated or high risk, would not be appropriate for TMH-V care (5/38, 13%). Finally, some noted an inability to conduct a thorough assessment, including a physical examination, using TMH-V (6/38, 16%).

The most frequently endorsed concern fell within the construct of effort expectancy and involved technological problems, including suboptimal audio and video quality, insufficient bandwidth to support videoconferencing, and malfunctions during treatment sessions (23/38, 61%). TMH-V was also often associated with increased workload and hassle due to factors such as equipment setup and additional scheduling processes (16/38, 42%). With respect to social influence, two studies (5%) mentioned the negative impact of having poor communication or support from leadership regarding TMH-V. In terms of facilitating conditions, several articles described a need for technical support and training (9/38, 24%), as well as having limited access to TMH-V equipment, clinic space, and funding (6/38, 16%).

### 3.2 | Attitude variability related to extent of TMH-V experience

#### 3.2.1 | Users versus nonusers

Several studies drew comparisons between users and nonusers of TMH-V within their samples. Compared to those with no experience, users had more positive attitudes toward TMH-V (Adler et al., 2014; Gilmore & Ward-Ciesielski, 2019) and expressed more confidence regarding their ability to provide care through TMH-V (Adler et al., 2014; Austen & McGrath, 2006). Providers with TMH-V experience were less likely to cite drawbacks to use such as staffing and credentialing concerns (Adler et al., 2014) or difficulties operating the technology, managing high-risk patients, or developing a therapeutic alliance (Interian et al., 2017). Predictors of increased use of TMH-V included finding the technology easy to use and having a history of training (Gibson et al., 2011; Glover et al., 2013; Simms et al., 2011) as well as having more years in practice (Gilmore & Ward-Ciesielski, 2019; Simms et al., 2011). A study of psychiatry residents found that those in rural as opposed to urban settings were more likely to plan on using TMH-V after residency (Glover et al., 2013). One study noted that younger age was related to TMH-V use (Gilmore & Ward-Ciesielski, 2019); however, more years in practice was also reported as predictive of TMH-V use, making this finding difficult to interpret given that these variables would likely be negatively correlated.

#### 3.2.2 | Preuse versus postuse

Although relatively few studies assessed providers’ attitudes before and after TMH-V use, the majority revealed an increase in positive sentiments with experience. Studies reported increases in positive opinions (Brooks et al., 2012; Whitten & Kuwahara, 2004), interest (Glover et al., 2013), comfort level (Gelber, 2001), and ease of use of the technology (Gibson et al., 2011), as well as decreases in providers’ skepticism (Elford et al., 2000) and apprehension (Brooks et al., 2012) following use. Several studies reported that providers were pleasantly surprised after trying TMH-V, noting that it was “much better than I thought it would be” (Elford et al., 2000), and caused less disruption to clinical care (Adler et al., 2014) and rapport development (Lindsay et al., 2017) than anticipated. Multiple studies noted that providers were surprised by their patients’ generally positive attitudes toward TMH-V (Adler et al., 2014; Brooks et al., 2012; Kopel et al., 2001; Newman, Bidargaddi, & Schrader, 2016), their willingness to participate, and the speed at which they adapted to this new mode of treatment delivery (Whitten & Kuwahara, 2004). In contrast, one qualitative study noted that two providers characterized TMH-V consultations as “twice as hard” to conduct as in-person sessions because of poor audio and video quality; they described being unable to detect nonverbal cues and needing to use shorter sentences and exaggerated gestures (Starling & Foley, 2006). However, it is worth noting that this study was published in 2006 and that the technology being evaluated is likely not representative of the current quality of TMH-V platforms.

Some providers with TMH-V experience noted that the benefits of TMH-V outweighed its various drawbacks and described the development of strategies and workarounds to address barriers encountered during use (Gibson et al., 2011; Lindsay et al., 2017; Simms et al., 2011). These included developing relationships with local law enforcement and hospitals to implement safety protocols in the case of a patient emergency, panning the camera around the provider’s treatment room to reassure wary patients that no one else was overhearing their confidential session, or completing the remainder of a session by phone if the video connection unexpectedly failed.

### 3.3 | Additional distinctions in providers’ attitudes

#### 3.3.1 | Provider satisfaction with TMH-V versus in-person care

Most studies comparing providers’ opinions toward TMH-V and in-person care found the latter to be more desirable. Providers reported preferring in-person care when completing child assessments and interventions (Elford et al., 2000; Levy & Strachan, 2013), and psychodynamic therapists described TMH-V as being slightly less effective than in-person sessions (Gordon et al., 2015). A minority of psychiatry residents with TMH-V experience felt that TMH-V was equal to face-to-face encounters (34%; 40% noted that it was not equal, and 26% were neutral; Glover et al., 2013). Providers conducted both TMH-V and in-person sessions in four intervention studies included in this review: a randomized controlled trial (RCT) of psychologists completing a cognitive behavioral therapy protocol for bulimia (Ertelt et al., 2011), an RCT of psychiatrists providing medication management and supportive counseling (Ruskin et al., 2004), a non-RCT in which psychologists conducted clinical intake interviews (Schopp, Johnstone, & Merrell, 2000), and a non-RCT in which psychiatrists provided school-based medication management and assessment to children (Mayworm et al., 2019). In-person sessions were rated significantly higher in terms of provider satisfaction (Mayworm et al., 2019; Ruskin et al., 2004; Schopp et al., 2000), as well as providers’ perception of goal formation, task completion, and development of a therapeutic bond (Ertelt et al., 2011). Three of these four studies shared that, despite being significantly lower than in-person scores, TMH-V ratings remained high (Ertelt et al., 2011; Mayworm et al., 2019; Ruskin et al., 2004). Several studies reported that providers found TMH-V to be adequate (Kopel et al., 2001), equivalent (Elford et al., 2001), or an acceptable alternative (Elford et al., 2000; Thomas et al., 2017) to in-person sessions, noting that there was “little to no difference” in care (Volpe et al., 2013). It is notable that these studies examined short-term consultation or assessment services delivered via TMH-V versus longer term psychotherapy or medication management.

#### 3.3.2 | TMH-V provider satisfaction as compared to patients and referring providers

Studies that compared provider and patient attitudes toward TMH-V found patients to be more satisfied than providers on average (Cruz, Krupinski, Lopez, & Weinstein, 2005; Shulman et al., 2017; Thomas et al., 2017). Providers rated working alliance measures as lower for TMH-V versus in-person sessions of a cognitive behavioral therapy protocol, while there were no significant differences in patient-rated scores between modalities (Ertelt et al., 2011). Another study reported that providers found technical difficulties to be more problematic and burdensome than patients; the authors posited that patients were more accustomed to experiencing delays when receiving health care as compared to providers (Schopp et al., 2000). In a study of emergency room providers who requested TMH-V consultation from a psychiatrist or social worker at a remote hospital, the providers requesting the patient consultation were more satisfied with the service than the providers conducting the TMH-V session (Thomas et al., 2017). It was hypothesized that the workflow of those providing TMH-V consultation was impacted more than the workflow of the requesting providers.

### 3.4 | Risk of bias

There is the risk of publication bias within the current review, such that negative or contradictory findings regarding providers’ attitudes toward TMH-V may have been omitted from results sections or may have precluded publication of completed research altogether. A risk of selection bias was identified within all included studies. Specifically, it is possible that the providers who opted to complete TMH-V surveys or participate in TMH-V intervention protocols may be categorically different from providers who opted out. Only one study (Shulman et al., 2017) reported the number of providers who elected to participate relative to the total number approached. Performance bias can occur within unblinded studies and refers to the provision of increased attention to the experimental group (i.e., those receiving TMH-V) as compared to the control group, beyond the scope of the actual intervention. Four studies assigned patients to TMH-V or in-person care (Ertelt et al., 2011; Mayworm et al., 2019; Ruskin et al., 2004; Schopp et al., 2000); providers in these studies may have given additional attention to patients in the TMH-V condition given the novelty of this modality, which could have impacted their subsequent perceptions of TMH-V effectiveness. Performance bias was also possible in the multiple other intervention studies without an in-person control condition, as providers may have put more effort into TMH-V sessions conducted within the context of a research study. Detection bias is also inevitable in this context, given that the main outcomes of this review are providers’ attitudes toward using TMH-V, preventing any sort of blinding regarding treatment condition when considering outcomes. Attrition bias is a significant concern among the included intervention studies. Only one study (Adler et al., 2014) reported on the observed attrition rate of therapists, with attrition here referring to inability to ultimately provide TMH-V care due to clinic and organization-level barriers. The remaining studies only included providers who participated in the full intervention within their samples, inviting the possibility that providers who ultimately dropped out of the intervention or were unable to provide TMH-V were not accounted for in analyses. Selective reporting bias is also likely given that many included studies did not clearly specify outcomes of interest within their analytic plans.

## 4 | DISCUSSION

The current systematic review revealed a diverse and growing literature examining providers’ attitudes toward TMH-V, and findings were positive overall. TMH-V was described as improving access to care for patients, increasing efficiency of services, saving time and money, and being more effective than in-person care in some circumstances. Additional benefits included TMH-V being easy to use, well-received by patients, supported by organizational leadership, and facilitated by strong training and technical support systems. While few articles reported negative overall attitudes toward TMH-V, many drawbacks to use were mentioned across studies. Technological difficulties were the most common concern and were reported in the majority of articles. Increased hassle and workload were also frequently mentioned. Most studies noted that TMH-V could feel impersonal, interfere with the therapeutic relationship, or impede the detection of nonverbal cues. Some providers reported beliefs that patients would not like TMH-V or that certain patients would not be appropriate to receive care remotely. Safety, security, liability, and confidentiality concerns were noted, as was the difficulty of conducting a thorough assessment via TMH-V. Lack of facilitating conditions, including training and technical support, was also endorsed. Poor support from leadership was a less frequently mentioned contributor to negative attitudes.

This finding that overall attitudes toward TMH-V were positive despite the presence of many drawbacks suggests that the relative weights of its benefits and disadvantages are not equal. Specifically, the perceived benefits of increasing efficiency, flexibility, and access to care for patients may offset the relative impersonality of TMH-V as well as its technological difficulties and increased provider burden. This finding aligns with the UTAUT, which posits that the performance expectancy of an innovation is the most significant predictor of its acceptance (Venkatesh et al., 2003).

Current findings also suggest a relationship between experience using TMH-V and providers’ attitudes. Specifically, providers with experience using TMH-V reported more acceptance of the modality than nonusers. A trend emerged in which providers experienced increases in positive sentiments upon using TMH-V, including being, “pleasantly surprised” by its functionality and ease of use (Adler et al., 2014; Elford et al., 2000; Lindsay et al., 2017). These findings, while tentative, align with UTAUT predictions, such that the perceived effort required to use an innovation declines following experience interacting with and adjusting to the technology (Venkatesh et al., 2003). In keeping with this theory, workflow modifications would become practiced over time and require less hassle on the part of the provider. Furthermore, experienced providers described developing strategies and workarounds to address barriers to use. This suggests that with experience, providers can mitigate some of the negative aspects of TMH-V, contributing to more positive overall attitudes toward the innovation.

However, while providers’ attitudes toward TMH-V tended to improve with experience, they still generally displayed a preference for conducting appointments in person versus via TMH-V. The exception to this finding involved short-term consultation and assessment procedures, in which TMH-V was deemed largely equivalent to in-person sessions. Therefore, TMH-V satisfaction may vary based on the nature of services being provided. For example, difficulties establishing a therapeutic alliance from behind a screen may have a larger impact within longer term care, in which the alliance develops over time and is an important predictor of therapy success (Martin, Garske, & Davis, 2000), as compared to brief patient interactions that do not involve follow-up. These difficulties may particularly impact psychologists, who may be more likely to have frequent and longer sessions with patients (e.g., weekly evidence-based treatment protocols) as opposed to providers primarily offering brief consultations or periodic medication management appointments. Findings emphasize the importance of optimizing audio and video quality to improve the ability to connect with patients via TMH-V, by increasing detection of subtle nonverbal cues and minimizing audio and video lags that can disrupt conversation flow. Through training, providers can also be made aware of these drawbacks and develop new strategies, for instance adjusting camera placement to ensure proper angles and lighting to best see and be seen by the patient, or maintaining a low threshold to inquire about recent substance use, given the greater difficulty of detecting intoxication remotely (Shore, 2013). That being said, there may be instances in which an in-person appointment is deemed necessary to conduct a thorough assessment (e.g., to measure vital signs if the patient does not have a home monitoring device such as a personal blood pressure cuff). This will likely be a greater barrier to prescribing clinicians or behavioral medicine specialists as opposed to those who do not conduct physical examinations as part of their treatment protocols.

Although TMH-V satisfaction levels were high overall, providers reported lower satisfaction than their patients. It is possible that the perceived drawbacks of establishing a therapeutic relationship via TMH-V may be more salient to providers than to patients, as noted in one study in which providers reported lower working alliance scores for TMH-V versus in-person sessions, while patient scores did not differ across modalities (Ertelt et al., 2011). The degree of effort required to use TMH-V also likely contributes to observed discrepancies in satisfaction. TMH-V represents a shift in the care model, such that providers are expected to reach out to their patients, versus their patients being expected to come to their office, a dynamic that redistributes effort expenditures and may in turn affect satisfaction. Indeed, TMH-V is more likely to impose burdens on providers—in terms of undergoing training and integrating new technologies and processes into their preexisting workflows—as compared to patients, for whom TMH-V is intended to decrease the more burdensome aspects of care. Furthermore, patients may be more accustomed to encountering delays and adapting to unfamiliar systems when receiving care as compared to their providers (Schopp et al., 2000).

Several aspects of the UTAUT were notably underrepresented within the reviewed articles. Although many articles discussed the moderating role of experience, there was little to no information shared regarding the role of gender, age, and voluntariness of use on providers’ acceptance of TMH-V. Future research should aim to examine these factors as they may influence attitudes toward TMH-V adoption. Indeed, findings of demographic differences within the broader mental health technology literature are complex and not consistent across studies (Glueckauf et al., 2018; McMinn, Bearse, Heyne, Smithberger, & Erb, 2011). There was also relatively little information provided regarding social influence from leadership or contextual factors. The role of organizational support can significantly influence providers’ attitudes, as discussed within established implementation frameworks (e.g., CFIR; Damschroder et al., 2009). Indeed, one study in the current review emphasized the importance of developing an organization-level “telehealth culture” involving information sharing across providers, policy development, training, and system-wide changes in administrative systems and staffing to ensure successful and sustained uptake of TMH-V (Newman et al., 2016).

Developing a TMH-V implementation plan, including formalized education, training, and supervision, as well as the involvement of experienced facilitators, can help to increase provider buy-in, enthusiasm, and confidence navigating this new technology (Fletcher et al., 2018). These strategies will be especially important to consider when moving beyond small-scale trials and toward the widespread integration of TMH-V into general practice. Ideally, successive generations of providers will receive TMH-V education and training as part of their graduate programs, which will increase the ubiquity of these services. However, postgraduate training is necessary to ensure that current providers gain comfort and experience navigating this technology and that these skills are maintained over time as technologies continue to evolve. It is notable that few of the reviewed studies described the nature and extent of TMH-V education and training received by providers. Future studies should prioritize examination of these factors and their influence on providers’ attitudes toward TMH-V.

Limitations to the current conclusions must be noted, particularly regarding the observed relationship between attitudes toward TMH-V and experience. Importantly, there is no way to determine the causality of provider sentiments; for instance, providers with more positive a priori opinions toward TMH-V or who experienced fewer barriers to use may have been more likely to ultimately use TMH-V. Alternatively, use of TMH-V may have subsequently increased providers’ positive opinions. Similarly, selection bias may exist in study designs; for instance, providers with more positive attitudes toward TMH-V may have been more likely to participate in the included studies, while providers who were unable to integrate TMH-V into their practice, who discontinued TMH-V use, or who had negative attitudes overall may not be adequately represented. In addition, many articles did not distinguish between providers with or without TMH-V experience when describing attitudes toward use. This represents a significant limitation as the potential moderating role of experience could not be examined, such as whether there was variation in the number or type of drawbacks endorsed by these groups. Furthermore, measures of providers’ attitudes and satisfaction with TMH-V were highly variable across studies as there is no “gold standard” measurement of these constructs, which prevents direct comparison of findings and limits overall interpretations. Additional limitations include the omission of non-English-language publications and the small sample sizes of some of the included studies. Indeed, the experience of implementing and using TMH-V within a small trial may be very different from attempts to integrate TMH-V into larger and more complex settings. Although a consensus approach was used to determine article inclusion, the systematic review analyses were conducted by the first author and did not involve additional formal consensus procedures, which could be considered a limitation. However, all co-authors provided critical feedback regarding the analytic plan and presentation of current findings. Finally, 12 of the included studies were published over 10 years ago, and given the time taken to conduct and publish research, many of the more recent studies are likely also referring to technologies that are now somewhat outdated. Given the fast-moving pace of innovation, it is possible that some of the drawbacks discussed in this review may be specific to older TMH-V equipment and processes that have since been refined. This in turn limits the potential generalizability of these findings to current conditions and emphasizes the need for ongoing review of the literature as TMH-V technology continues to evolve and improve.

Although the current review focused on TMH-V, it is important to note that additional technologies may fall within the domain of telemental health, including e-mail, smartphone apps, message boards, or web-based therapy protocols. Two of the studies included in the current review surveyed providers’ attitudes toward telemental health technologies more broadly, with only TMH-V results reported here (Cipolletta & Mocellin, 2018; Glueckauf et al., 2018). Future work should aim to synthesize findings from papers that evaluate multiple forms of telemental health concurrently, in order to examine variation in provider attitudes based on mental health technology type. Future studies should also examine whether there may be discipline-level variability in providers’ attitudes toward TMH-V that may relate to differences in typical encounter characteristics; for instance, whether prescribing clinicians with less frequent and shorter appointments with their patients may have more positive views toward TMH-V than psychologists with longer and more frequent sessions.

The finding that providers’ positivity toward TMH-V may have outweighed various barriers to use could have important implications when attempting to implement this technology in practice. If providers believe that using TMH-V will improve overall access to care and ultimately increase efficiency and flexibility, they may be more willing to accept initial increases in hassle and disruptions in workflow, as well as an altered quality of connection with patients. This distinction is important to consider when introducing TMH-V to providers, specifically preparing providers for the inevitable growing pains of adopting this new technology, but emphasizing the net benefits that TMH-V may provide to their patients and themselves with sustained use.

The balance between perceived benefits and drawbacks of TMH-V among providers is also likely to continually shift in response to rapid advances occurring in the field. Improvements in the quality of communication via TMH-V, due to strengthened broadband connectivity and increasingly sophisticated video and audio technologies, may help to lessen the relative disadvantages of connecting with a patient from behind a screen (Brooks et al., 2013). With continued use and troubleshooting, TMH-V will ideally become more seamlessly integrated into electronic health record, scheduling, and billing platforms, which would relieve a substantial amount of staff burden. In addition, the introduction of legislation allowing for the wider provision of telehealth, such as a recently enacted bill allowing TMH-V to occur across state lines within the Veterans Health Administration, as well as similar legislation within the Department of Defense, will decrease logistical barriers to care (Brooks et al., 2013; U.S. Department of Veterans Affairs, 2018; Zur, 2019). State laws requiring insurers to reimburse TMH-V appointments to the same extent as in-person care (i.e., parity laws) will also be critical in increasing uptake, as will the relaxation of mandates in some states requiring out-of-state providers to obtain special licenses to provide telehealth services (Adler-Milstein, Kvedar, & Bates, 2014). In fact, a Psychology Interjurisdictional Compact (PSYPACT), which would provide a streamlined certification process for psychologists to conduct TMH-V across state lines, is currently in development (Wicklund, 2019b); similar compacts have already taken effect for physicians and nurses across multiple states (Wicklund, 2019a). The development and dissemination of established clinical competencies and guidelines for care will also likely help to standardize TMH-V practices and increase provider confidence (Hilty, Maheu, Drude, & Hertlein, 2018; Shore et al., 2018). Furthermore, providers’ comfort interacting with patients via TMH-V may continue to increase as videoconferencing becomes ubiquitous across multiple domains, from coordinating remote work meetings to connecting with family members using smartphone video chat features.

It is also worth noting that many of the most unique strengths of TMH-V involve providing services directly to patients’ homes versus to remote clinics—for instance allowing immobile and severely ill patients to receive care, providing a window into a patients’ living environment, and serving as a stepping stone into services for avoidant or highly anxious patients (Fletcher et al., 2018; Gordon et al., 2015; Lindsay et al., 2017). Advancements in technology, including improved wireless connectivity and facilitated access to smartphones and tablets, will allow more TMH-V to take place within patients’ homes, which may further increase the relative advantages of this modality. Only five of the included studies in this review delivered TMH-V to patients’ homes, and four of those studies were published in the past three years, highlighting the relative novelty of this modality and the need for additional research to understand possible effects of TMH-V delivery location (e.g., clinic or home) on provider and patient perceptions as well as clinical outcomes.

Yet importantly, current findings reveal that providers prefer in-person contact when given the choice. It therefore remains likely that regardless of future advancements in technology, in-person contact will continue to be preferred by many providers and patients when it is easily accessible. As such, TMH-V can be viewed as one of many effective treatment modalities whose relative advantage will vary depending on the unique circumstances of each clinical contact. For some patients with significant barriers to receiving in-person care, TMH-V technology may represent the first and only way in which they are able to undergo therapy. Even if a provider may generally prefer in-person appointments when given the choice, the benefit of providing therapy via TMH-V to a patient who otherwise would receive none is clear. A hybrid model of care may be ideal in other cases, such that patients receive a combination of in-person and TMH-V sessions based on changing needs over the course of therapy (Yellowlees & Nafiz, 2010). These hybrid care structures will likely become more common as TMH-V uptake increases, and their use and effectiveness should be studied.

In sum, the current review revealed that providers’ attitudes toward TMH-V are positive overall, despite the acknowledgment of multiple drawbacks to its use. This finding suggests that the relative advantages of TMH-V in certain circumstances, such as increasing access to care where services are limited, may outweigh its various disadvantages from the provider perspective. Gaining experience conducting TMH-V sessions with patients may lessen perceived drawbacks of use and foster the development of strategies and workarounds to improve care delivery. As health-care systems increasingly prioritize TMH-V, it will be crucial to consider providers’ attitudes and perspectives when working to facilitate uptake, as they will play a key role in the successful implementation of this innovation.

## Figures and Tables

**TABLE 1:** Summary of articles included in systematic review (*N* = 38)

References	*N*	Providers sampled and TMH-V experience	TMH-V services provided	Patients served	Location of patients’ TMH-V care	Study design and/or provider measures *(Additional collected data not analyzed in current review included in italics)*	Main findings
1. Adler et al. (2014)	12	Psychologists, social workers, and counselors; 2 of the 12 clinicians ultimately provided TMH-V	Psychotherapy	Veterans in South Central United States	VA community-based outpatient clinics	Pre–post surveys of providers who both did and did not engage in TMH-V intervention	Adopters had more positive views of TMH-V at preassessment and nonadopters endorsed more barriers. Adopters noted increases in knowledge, confidence, and motivation at postassessment. Intervention was less disruptive than initially imagined
2. Austen and McGrath (2006)	134	Nurses, psychologists, and psychiatrists; 12% had TMH-V experience	Psychotherapy and/or medication management	Deaf and nondeaf patients in the UK	Unknown or not applicable	Questionnaire	Those with experience using TMH-V felt more confident regarding their abilities to use the technology. Providers report relatively low access to TMH-V technologies
3. Baird et al. (2018)	83	Psychiatric advanced practice nurses; 63% had TMH-V experience	Telenursing	Children and adults in the United States	Unknown or not applicable	Online survey	Attitudes toward TMH-V were positive overall, and providers would like more training
4. Brooks et al. (2012)	39	Unspecified clinicians involved in developing 3 TMH-V clinics	Psychotherapy and/or medication management	Native American Veterans in the Northern Plains	VA community-based outpatient clinics	Semi-structured phone interviews *Implementation process and timeline data also collected*	Positive impressions of TMH-V increased over time from 67% to 82%, due to providers gaining experience and receiving positive feedback from patients and staff
5. Cipolletta and Mocellin (2018)	289	Psychologists; 8.3% had TMH-V experience	Psychotherapy	Patients in Italy	Unknown or not applicable	Anonymous survey *E-mail, text, and online forum data also collected*	62.6% were favorable toward TMH-V
6. Cruz et al. (2005)	4	Psychiatrists, psychologist; all provided TMH-V care	Psychotherapy, medication management, and/or consultation	Children, adolescents, and adults in Arizona	Referring rural hospitals with no mental health providers	Satisfaction form completed after each patient contact *Patient data (satisfaction, diagnoses, demographics, # of visits) also collected*	Providers noted that TMH-V improved clinical efficiency for 61% of appointments, but were generally less satisfied with TMH-V and endorsed more barriers than their patients
7. Cunningham et al. (2013)	10	Psychiatrists, social workers, and counselors; all provided TMH-V care	Consultation	Schoolchildren in urban Maryland	8 schools	Anonymous online survey	Providers reported positive experiences with TMH-V, rated comfort using technology as 9.75 out of 10 and described process as efficient
8. Elford et al. (2000)	5	Psychiatrists; all provided TMH-V care	Diagnostic assessments	Children and adolescents in Newfoundland	Child psychiatry center within a hospital	Pre–post surveys of TMH-V intervention, satisfaction questionnaires after each assessment *Also measured agreement between TMH-V and in-person diagnoses and patient/parent satisfaction*	21 of 23 sessions rated as going moderately well or very well. All psychiatrists endorsed TMH-V as acceptable alternative to in-person sessions, but they prefer in-person and feel it allows for better communication. Noted fewer barriers and less skepticism toward TMH-V at follow-up compared to baseline
9. Elford et al. (2001)	5	Psychiatrists; all provided TMH-V care	Diagnostic assessments	Children and adolescents in Newfoundland	Child psychiatry center within a hospital	Satisfaction questionnaires after each assessment *Patient/parent satisfaction and cost data also collected*	All 25 assessments rated as satisfactory or very satisfactory. 21 were rated as equivalent to in-person, 3 as not as good but “good enough,” and 1 as superior to in-person due to ability to zoom camera on facial tic for diagnostic purposes
10. Ertelt et al. (2011)	6	Psychologists; all provided both TMH-V and in-person care	Cognitive behavioral therapy for bulimia	Adults with bulimia diagnoses in North Dakota and Minnesota	Distal therapy sites	RCT comparing TMH-V to in-person treatment. Providers completed Working Alliance Inventory (WAI) questionnaire *Patient WAI data also collected*	Providers rated adherence to therapeutic tasks, goals, and therapeutic bond significantly higher for in-person versus TMH-V sessions; TMH-V means were 1–2 points lower than in-person. No significant differences in patient ratings of in-person and TMH-V sessions
11. Gelber (2001)	25	Unspecified clinicians; all delivered TMH-V care	Consultation and other clinical work	Children and adolescents in rural Australia	CAMHS clinics	Telephone survey *Length and frequency of use data also collected*	50% valued TMH-V use and 45% valued highly. 96% reported an increased comfort level over time, described adapting to the technology
12. Gibson et al. (2011)	68	Psychologists, psychiatrists, social workers, and nurses; 49% had TMH-V experience	Consultation	Patients in remote and rural First Nations communities in Canada	Community centers	Online survey of all providers and qualitative interviews of those with TMH-V experience *Frequency of use data also collected*	50% of survey respondents rate TMH-V as useful, 9% rate as not useful at all. Those who rated TMH-V as easier and more useful and who underwent training were more likely to use TMH-V more often. TMH-V is described as becoming easier to use with more experience
13. Gilmore and Ward-Ciesielski (2019)	52	Masters and PhD level psychotherapists; 50% had TMH-V experience	Psychotherapy	Patients with acute suicide risk in the United States	Unknown or not applicable	Online survey	Providers who had more positive attitudes toward TMH-V and had more years in practice were more likely to use TMH-V with patients at high risk for suicide
14. Glover et al. (2013)	283	Psychiatry residents; 18% had TMH-V experience	Medication management	Children, adolescents, and adults in the United States	Unknown or not applicable	Online survey *Frequency of use data also collected*	72% were interested/very interested in TMH-V. 72% of those with prior experience said that their interest in TMH-V increased with use. 40% said TMH-V is not equal to in-person care, while 34% felt it is equal
15. Glueckauf et al. (2018)	164	Psychologists; 26% had TMH-V experience	Psychotherapy	Children, adolescents, and adults in the United States	Unknown or not applicable	Anonymous online survey *Telephone, text, e-mail, and frequency of use data also collected*	73% describe TMH-V as useful
16. Gordon et al. (2015)	176	Psychologists, psychiatrists, and social workers; 79% had used TMH-V for >3 years	Psychodynamic psychotherapy	Students in China	Patient’s home	Online survey *Attitudes toward teaching and supervision via telehealth also measured*	TMH-V rated as “slightly less effective” than in-person care on factors such as symptom reduction, privacy, exploring transference and countertransference, and relational problems
17. Interian et al. (2017)	33	Psychologists, psychiatrists, social workers, and nurses; 61% had TMH-V experience	Psychotherapy	Urban, suburban, and rural US veterans	Patient’s home	Semi-structured interviews *Implementation process and rate of uptake data also collected*	Those with no TMH-V experience more consistently questioned the effectiveness of TMH-V as compared to current users. Current users noted satisfaction with TMH-V but also encountered significant logistical barriers
18. Jameson et al. (2011)	86	Psychologists, psychiatrists, social workers, and therapists; 58% had TMH-V experience	Medication management and psychotherapy	Urban and rural veterans in southern United States	VA community-based outpatie	Semi-structured interviews and phone surveys *Utilization data also collected*	Effectiveness scores for diagnostic interviews and psychotherapy were positive. Providers wanted to see research comparing TMH-V to in-person effectiveness, noted loss of in-person contact and technical issues as barriers
19. Kopel et al. (2001)	8	Psychiatrists; all providers delivered TMH-V care	Assessment and consultation	Children and adolescents in rural New South Wales	Mental health clinics	Technology evaluation questionnaire completed after each assessment *Patient and parent satisfaction data also collected*	79% of sessions rated as adequate compared to in-person, 15% almost as good, 4% poor, and 1% rated as good as in-person. Ease of use rated as fair at 47% of sessions and good or excellent at 49% of sessions. Providers surprised how positively families responded to TMH-V
20. Levy and Strachan (2013)	61	Medical, nursing, and psychology staff; 62% had TMH-V experience	Assessment and intervention	Children and adolescents in rural Scotland	CAMHS clinics	Online and paper surveys	Majority think TMH-V would improve local access and are willing to introduce it into their service, but most would prefer in-person care
21. Lindsay et al. (2017)	5	Psychologists, social workers, counselors, and psychology interns; all delivered TMH-V care during intervention	Psychotherapy	Veterans in rural Mississippi	Patient’s home	Qualitative phone interviews *Implementation process and uptake data also collected; qualitative interviews also conducted with patients*	Overall satisfaction with TMH-V modality. Providers noted multiple barriers to use but described being flexible and adapting following unforeseen technological issues
22. Mayworm et al. (2019)	7	Psychiatrists; all conducted TMH-V and in-person sessions	Medication management and assessment	Schoolchildren in Baltimore	25 schools	Anonymous satisfaction surveys, focus groups *Patient, caregiver, and referring clinician satisfaction data also collected; efficiency analyses also conducted*	Providers rated satisfaction with TMH-V as 4 out of 5. Note increased access to care and flexibility. Ease of use rated lower. Providers preferred in-person sessions but satisfaction rates were similar between modalities
23. Mitchell et al. (2009)	19	Psychologists, psychiatrists, social worker, and nurse; all with TMH-V experience	Direct clinical care	Children and adolescents in Scotland	CAMHS clinics and hospitals	Questionnaires	79% prefer TMH-V over telephone communication. More benefits of TMH-V were noted as compared to drawbacks
24. Monthuy-Blanc et al. (2013)	205	Psychologists, social workers, nurses, natural helpers; none had TMH-V experience	Psychotherapy	First Nations communities in Canada	Unknown or not applicable	Technology Acceptance Questionnaire	The only significant predictor of providers’ intention to use TMH-V was its perceived usefulness
25. Moreau et al. (2018)	40	Psychologists, social workers, primary care providers; some had TMH-V experience	Psychotherapy	Female veterans in urban and rural Midwest and southern United States	VA facilities	Semi-structured qualitative interviews	Providers enthusiastic about using TMH-V to improve access to care for female veterans. Noted multiple barriers including technology challenges and need for safety protocols
26. Newman et al. (2016)	>40	Psychiatrists, nurse practitioners; some had TMH-V experience	Psychotherapy and assessment	Patients in rural Australia	Hospitals and clinics	Phone interviews and focus groups *Utilization data also collected*	TMH-V accepted to varying extents across providers, with many citing its ability to increase access. Multiple drawbacks noted as well as a need for further training and development of “telehealth culture”
27. Perle et al. (2014)	782	Psychologists and trainees; 19.4% had TMH-V experience	Psychotherapy	Patients in the United States and Canada	Unknown or not applicable	Online surveys	79.5% agreed that TMH-V can be effective treatment; fewer (58.3%) felt TMH-V to patient’s home would be effective. 42% unsure whether TMH-V is as effective as in-person care
28. Pesämaa et al. (2007)	26	Psychiatrists; all had TMH-V experience	Consultation	Children and adolescents in Finland	Hospitals	Questionnaire *Utilization data also collected*	All providers agreed that TMH-V saves time, costs, and work; 35% agreed that it improves the quality of services. Multiple technological barriers to use noted
29. Ruskin et al. (2004)	8	Psychiatrists; all provided both TMH-V and in-person care	8 sessions of medication management and supportive counseling over 6 months	Veterans in Maryland	VA facility	RCT comparing TMH-V to in-person treatment. Satisfaction questionnaire completed at week 26 *Patient satisfaction, adherence, treatment outcome, and cost data also collected*	Psychiatrist satisfaction was significantly greater for in-person sessions versus TMH-V. However, satisfaction ratings were high in both conditions, suggesting positive perception of TMH-V
30. Schopp et al. (2000)	9	Psychologists and trainees; all conducted TMH-V and in-person sessions	Clinical interviews	Adults in rural midwestern United States	Hospitals and clinics	Satisfaction questionnaire after each in-person or TMH-V session, and qualitative interviews *Patient satisfaction and cost data also collected*	Provider satisfaction was significantly higher for in-person versus TMH-V sessions. Providers described greater frustration with technological delays as compared to patients
31. Shulman et al. (2017)	31	Psychiatrists; 6 of whom ultimately provided both TMH-V and in-person care	Medication management and psychotherapy	Patients at a New York hospital outpatient psychiatry clinic	Patient’s home	RCT comparing in-person to TMH-V psychiatric care; providers completed online survey *Patient satisfaction and adherence data also collected*	Authors reported difficulty recruiting providers; those who agreed only selected a fraction of their patients as appropriate for TMH-V care and reported concerns about technical problems and extra hassle. Patients had more positive opinions of TMH-V experience than providers
32. Simms et al. (2011)	185	Psychologists, psychiatrists, social workers, and nurses; 40% had TMH-V experience	Psychotherapy	Patients, including Veterans, in Canada	Unknown or not applicable	Online survey and qualitative interviews	Majority rated TMH-V as very useful or somewhat useful, but more rated TMH-V as difficult to use as compared to easy. Those using TMH-V more frequently had more years in practice, more training, and perceived technology as useful and easy. Discussed barriers such as safety concerns and noted developing solutions
33. Starling and Foley (2006)	27	Psychologists, psychiatrists, and social workers; all participated in TMH-V intervention	Consultation	Children and adolescents in rural New South Wales	Clinics	Questionnaire	53% rated TMH-V as effective. Two providers reported it was “twice as hard” to conduct TMH-V sessions versus in-person due to difficulties engaging families, having to use shorter sentences and less nonverbal communication
34. Thomas et al. (2017)	148	Psychiatrists and social workers; all providers delivered TMH-V care	Psychiatric emergency consultation	Children and adolescents in Colorado	Emergency departments	Telehealth satisfaction instrument completed after each consultation *Utilization and caregiver/referring provider satisfaction data also collected*	Providers rated TMH-V as acceptable and rated ease of use and quality of care positively. Provider satisfaction scores were lower than those of referring providers and caregivers, likely due to increased workload, concerns regarding developing therapeutic alliance, and making accurate diagnoses
35. Volpe et al. (2013)	36	Psychiatrists, 83% had TMH-V experience	Consultation and short-term follow-up	Children and adolescents in rural Ontario, Australia, and the United States	Mental health clinics and ho	Online survey of 26 providers, focus groups, and qualitative interviews with 10 providers with TMH-V experience	68% of survey respondents described TMH-V as an important innovation providing increased access to care. 40% of survey respondents and majority of interviewees endorsed little to no differences between TMH-V and in-person consultations
36. Wagnild et al. (2006)	11	Psychiatrists; all had TMH-V experience	Medication management	Children, adolescents, and adults in rural Montana	Hospitals and clinics	Semi-structured interviews	Four of 11 providers reported satisfaction with TMH-V. Agreed that TMH-V improves access but cited many barriers including technology issues, difficulty establishing rapport, and trouble reading nonverbal cues
37. Whitten and Kuwahara (2004)	36	Psychiatrists; all participated in TMH-V intervention	Consultation	Children, adolescents, and adults in rural and urban Michigan	Clinic, youth center, crisis h homes	Pre–post focus groups and interviews during project implementation period *Utilization and patient satisfaction data also collected*	Majority of providers either started project with positive attitude toward TMH-V or developed positive attitude during participation; 1 provider reported negative attitude toward TMH-V before and during implementation. Majority were reluctant to initiate TMH-V but were pleasantly surprised by level of TMH-V acceptance by their patients
38. Wynn et al. (2012)	11	Psychologists and psychiatrists; none had TMH-V experience	Consultation	Patients in Norway	Not applicable	Qualitative interview	Providers were in general positive toward TMH-V given that they could first meet in-person. Had multiple concerns regarding potential effectiveness, technological difficulties, lack of training, and trouble developing rapport

Abbreviations: CAMHS, Child and Adolescent Mental Health Services; RCT, randomized controlled trial; TMH-V, telemental health via videoconferencing.

**TABLE 2:** Positive aspects of TMH-V generated by providers

UTAUT constructs and author-derived subconstructs^a^	Article frequency (percentage)^b^	Included articles^c^
**Performance expectancy**		
Increased access to care	16 (42)	5, 7, 12, 16, 17, 20, 21, 22, 23, 25, 26, 32, 34, 35, 36, 37
Saves time and money, efficient	12 (32)	5, 6, 7, 11, 20, 21, 22, 23, 26, 28, 32, 35
Can be more effective than in-person care	8 (21)	5, 7, 12, 16, 20, 21, 26, 37
Patients like TMH	6 (16)	1, 4, 7, 16, 19, 37
Increased flexibility	4 (11)	7, 21, 22, 35
New opportunities for provider	3 (8)	3, 7, 35
**Effort expectancy**		
Easy to use	7 (18)	1, 8, 12, 19, 28, 34, 35
**Social influence**		
Organization supportive of TMH	3 (8)	4, 26, 35
**Facilitating conditions**		
Availability of good technical support	4 (11)	4, 12, 17, 35

Abbreviations: TMH-V, telemental health via videoconferencing; UTAUT, unified theory of acceptance and use of technology.

a UTAUT constructs are bolded, and author-derived constructs are unbolded.

b Frequency and percentage of articles that included the given subconstruct, total *N* = 38.

c Numbers correspond to article numbers assigned in Table 1.

**TABLE 3:** Negative aspects of TMH-V generated by providers

UTAUT constructs and author-derived subconstructs^a^	Article frequency (percentage)^b^	Included articles^c^
**Performance expectancy**		
Impersonal/interferes with therapeutic relationship	19 (54)	3, 5, 6, 8, 9, 10, 11, 12, 13, 18, 20, 21, 28, 31, 32, 33, 34, 36, 38
Safety and legal concerns	13 (37)	3, 4, 5, 8, 12, 13, 16, 21, 25, 27, 31, 32, 35
Patients will not like TMH	8 (23)	1, 4, 17, 26, 31, 32, 36, 38
Security and confidentiality concerns	7 (20)	3, 5, 20, 27, 35, 36, 38
Not appropriate for certain patients	5 (14)	12, 21, 26, 32, 36
Unable to conduct thorough assessment	6 (16)	8, 13, 22, 34, 36, 38
**Effort expectancy**		
Technological problems	23 (66)	4, 5, 6, 7, 8, 9, 11, 12, 17, 18, 19, 20, 21, 25, 26, 28, 30, 31, 32, 35, 36, 37, 38
Increased work and hassle	16 (46)	1, 3, 4, 8, 17, 20, 21, 23, 26, 27, 31, 32, 33, 36, 37, 38
**Social influence**		
Poor communication or support from leadership	2 (6)	1, 17
**Facilitating conditions**		
Need for technical support and training	9 (26)	1, 3, 11, 20, 25, 28, 35, 36, 38
Limited space, equipment, and funding	6 (17)	1, 11, 12, 23, 25, 28

Abbreviations: TMH-V, telemental health via videoconferencing; UTAUT, unified theory of acceptance and use of technology.

a UTAUT constructs are bolded, and author-derived constructs are unbolded.

b Frequency and percentage of articles that included the given subconstruct, total *N* = 38.

c Numbers correspond to article numbers assigned in Table 1.

## Public Health Significance

Mental health providers can conduct appointments remotely via videoconferencing, a form of telemental health (TMH-V) which can increase access to care for patients who live far from specialty providers or have difficulty leaving their homes. It is important to understand providers’ attitudes toward this technology, as they play a crucial role in whether this service will be widely adopted by health-care systems and will therefore be offered to patients. The current review found that providers feel positive toward TMH-V despite reporting multiple drawbacks; this suggests that the relative advantages of TMH-V, such as its ability to increase access to care, may outweigh its disadvantages, including technological problems, increased hassle, and perceptions of feeling impersonal.
